# Design and characterization of phosphonic acid-functionalized grafted sepiolite nanohybrids and their adsorption studies for removal of copper ions from aqueous solution

**DOI:** 10.55730/1300-0527.3674

**Published:** 2024-01-09

**Authors:** Mehwish TAHIR, Asıf RAZA, Amara NASIR, Tariq YASIN, Shamila IMTIAZ

**Affiliations:** 1 Department of Chemistry, Pakistan Institute of Engineering and Applied Sciences (PIEAS), Islamabad, Pakistan; 2 Department of Chemical Engineering, Pakistan Institute of Engineering and Applied Sciences (PIEAS), Islamabad, Pakistan; 3 Department of Physics, Pakistan Institute of Engineering and Applied Sciences (PIEAS), Islamabad, Pakistan; 4 Department of Chemistry, Pakistan Institute of Engineering and Applied Sciences (PIEAS), Islamabad, Pakistan; 5 Chemistry Division, Directorate of Science, Pakistan Institute of Nuclear Science and Technology (PINSTECH), PO Nilore, Islamabad, Pakistan

**Keywords:** Nanohybrids, sepiolite, glycidyl methacrylate, free radical graft polymerization, phosphonation, adsorption

## Abstract

In this study, we synthesized novel, economically efficient phosphonic acid-functionalized grafted sepiolite nanohybrids for selective elimination of copper ions from water. These nanohybrids were prepared by graft polymerization of glycidyl methacrylate onto sepiolite. We utilized free radical graft polymerization to graft glycidyl methacrylate (GMA) onto silanized sepiolite. The nanohybrids obtained exhibited a grafting percentage of 479% at 0.3 g of KPS initiator, 15% GMA monomer, and after 4 h of reaction. In pursuit of selectively removing metal ions from water, the nanohybrid with the highest grafting (PGE_3_) was chemically treated with phosphoric acid to introduce phosphonic acid groups on it. FTIR, XRD, SEM, CHO analysis, BET, and TGA analysis were utilized to characterize the developed nanohybrids. Batch adsorption studies were carried out using AAS process, examining the impact of pH, adsorbent weight, contact time, adsorbate concentration, and temperature on the adsorption process. Due to the selectivity of phosphonic acid groups towards copper ions, phosphonic acid-functionalized grafted sepiolite nanohybrid (PGE_3_-P) was used for copper ions removal from its aqueous solution. The maximum adsorption capacity of PGE_3_-P adsorbents was 134.5 mg/g for copper ions. The data from kinetic studies suggests that the adsorption process of copper ions followed a pseudosecond-order model. Furthermore, Langmuir isotherm proved to be a more fitting model in equilibrium isothermal investigations. The thermodynamic analysis of the data indicates that the adsorption of copper ions by PGE_3_-P is an endothermic and spontaneous process. The development of this phosphonic acid-functionalized grafted sepiolite nanohybrid adsorbent is a new contribution into the field of adsorption. The developed material can be utilized as selective adsorbent for elimination of other heavy metals from water.

## 1. Introduction

The rapid increase in industrialization has led to the contamination of clean water streams with hazardous metals like copper, cadmium, chromium, nickel, lead, and mercury [1]. Industrial processes often involve the use and release of these metals into water streams, either intentionally or as byproducts of manufacturing activities. For example, copper is commonly used in electronic and electrical components [2], while cadmium is found in batteries [3]. Chromium is used in metal plating and other industrial processes [4], and mercury is present in certain chemical applications [5]. As these metals find their way into water streams, they pose serious threats to the environment and human health above their permissible limit. They can accumulate in aquatic ecosystems, contaminating drinking water sources, and adversely affecting aquatic life. Exposure to these metals can lead to a range of health issues in humans, from gastrointestinal problems to more severe conditions like damage to the nervous system, liver, and kidneys [6]. According to the U.S. Environmental Protection Agency (USEPA), the permissible limit of copper in industrial discharge is 1.3 mg/L. Water resources polluted with copper can cause nausea, diarrhea, vomiting and gastrointestinal problems in humans. Prolonged exposure may also result in hepatic necrosis, potentially leading to fatal outcomes [7]. To address these concerns, there is a need for effective water treatment and remediation strategies to remove or reduce the concentration of these hazardous metals in water. This is crucial for safeguarding both the environment and public health.

GMA is a versatile monomer. Its versatility arises from its chemical structure comprising a reactive epoxy group and a methacrylate group. This dual functionality renders GMA highly adaptable for a multitude of polymerization reactions [8]. It has gained recognition for its efficacy in both selective removal [9] and recovery of metals [10]. Its epoxy group can be converted into suitable functional groups for selective metal ion uptake [11]. Wang et al. synthesized triethylene teramine (TETA)-functionalized magnetic poly(glycidyl methacrylate) (PGMA) nanoadsorbents for effective adsorption of Hg (II) [12]. Zhao et al. developed a novel adsorbent by functionalizing poly(glycidyl methacrylate) microspheres with pyromellitic acid and used it to adsorb Pd (II) from wastewater [13].

The process of graft polymerization is an attractive method for creating nanohybrids, involving the polymerization of various monomers on a compatible substrate [14]. In such a process, the polymer grafting percentage is crucial and depends on reaction parameters like initiator amount, monomer concentration, and reaction duration. Therefore, optimizing these parameters is essential to obtain an optimum polymer grafted product [15]. Furthermore, modifying/functionalizing the substrate before polymer grafting is a unique strategy to increase the polymer grafting percentage on the substrate [16]. For instance, silanization of substrate by silane coupling agents has been used to modify the surface chemistry of substrate and thereby to increase the quantity of grafted polymer [17]. Nasir et al. polymerized acrylonitrile and aniline on a silanized GO substrate, resulting in a doubling of the grafting percentage for polyacrylonitrile [18] and a 1.5-fold increase for polyaniline compared to pristine GO, respectively [19].

In this study, we synthesized a GMA-grafted sepiolite nanohybrid using a free radical graft polymerization method. This nanohybrid serves as a precursor for the development of selective metal ion adsorbents. We utilized a silanized sepiolite substrate to graft GMA and optimized the reaction conditions, including initiator amount, monomer concentration, and reaction time. The optimal grafted product was further converted into phosphonic acid-functionalized grafted sepiolite nanohybrid through treatment with phosphoric acid. These developed nanohybrids were characterized using spectroscopic techniques such as FTIR spectroscopy, XRD analysis, TGA analysis, SEM, and BET analysis. The phosphonic acid-functionalized grafted sepiolite nanohybrid was employed for metal ion removal, utilizing Cu (II) as model pollutant.

## 2. Materials and methods

### 2.1. Materials

In the present work, pristine sepiolite (PS) (with 99% pure mineral contents), vinyl trimethoxy silane (VTMS, 97%), tween-80 (98%), glycidyl methacrylate (GMA) (97%, d = 1.042 g/mL), acetone (99.5%), isopropanol (>99.8%), methanol (99.7%), copper sulfate (99.9%), lead acetate trihydrate (99.9%), hydrochloric acid (37%), sulfuric acid and phosphoric acid were procured from Sigma-Aldrich, Germany. Potassium persulfate (KPS) purchased from Daejung, Korea, was used after recrystallization in water. All the chemicals were used as received.

### 2.2. Purification and silanization of sepiolite

The purification process for PS involved stirring it in deionized water for 24 h. Following this, the resulting product was filtered and subjected to vacuum drying. The dried product was then pulverized into fine powder and stored in a desiccator. This purified and dried PS was further utilized for reaction with VTMS [20]. In this study, VTMS was employed as a vinyl-based silane coupling agent due to its highly reactive nature [21]. In short, an appropriate quantity of PS was introduced into isopropanol in a round bottom flask and blended with hydrolyzed VTMS at room temperature. Afterward, the temperature was raised to 60 ºC ± 2, and the system was maintained under persistent stirring for a period of 2 h. The obtained product was filtered, washed with methanol, and then subjected to vacuum drying for 15 min. Subsequently, the dried product was pulverized, stored in a desiccator, and labelled as vinyl-modified sepiolite (VMS).

### 2.3. Free radical graft polymerization PGMA onto VMS

In free radical graft polymerization of PGMA on VMS, appropriate quantity of VMS, tween-80, and distilled water were added into a glass reactor. After stirring for 15 min, a stable suspension was obtained. GMA followed by KPS was then introduced into this suspension and the temperature was raised to 70 ºC for 4 h under an inert atmosphere. Subsequently, the resulting product was filtered, separated from homopolymer by Soxhlet extraction in DMF, dried, and labelled as PGE. The chemical compositions and codes of the prepared samples are shown in Table 1.

### 2.4. Grafting percentage

Grafting percentage is the amount of polymer that has been grafted on a base material. It is determined by measuring the increase in weight of the base material following grafting reactions. Typically, it is calculated using the following equation:


$$\text{Grafting percent }(\%)=\frac{{\text{W}}_{\text{g}}-{\text{W}}_{\text{o}}}{{\text{W}}_{\text{o}}}\times 100$$

where W_o_ = weight of PS before grafting, and W_g_ = weight of grafted product.

### 2.5. Phosphonation

**Figure 1:**
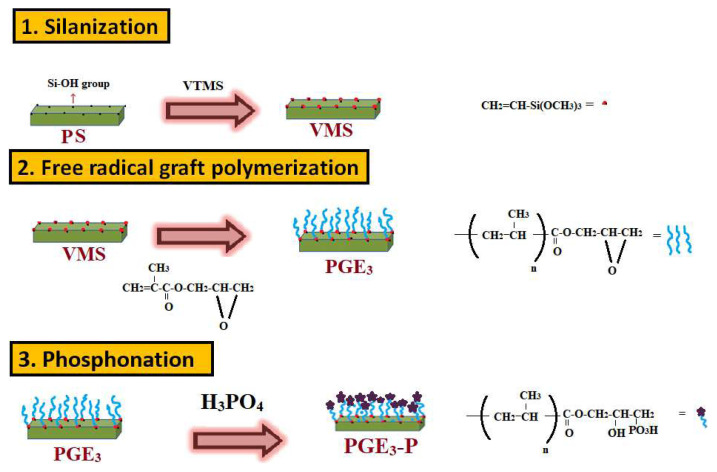
Key steps involved in synthesizing phosphonic acid-functionalized grafted sepiolite nanohybrid.

The epoxide groups of PGE_3_ were converted into phosphonated form. One gram of PGE_3_ was added to a two-necked round bottom flask, followed by addition of 10 mL of concentrated H_3_PO_4_. The mixture was stirred for 6 h at 80 °C. The resulting product, PGE_3_-P, was rinsed with distilled water for neutralization, followed by vacuum drying and storage in a desiccator for further use. The key steps involved in the synthesis phosphonic acid-functionalized grafted sepiolite nanohybrid are illustrated in Figure 1.

### 2.6. Characterization

The structural variations of the nanohybrids were analyzed using a Nicolet 6700 FTIR spectrophotometer with a diamond crystal. Scanning of all samples was conducted from 4000 to 400 cm^−1^ at a resolution of 6 cm^−1^. XRD analysis of all samples was performed at room temperature using a D8 Discover X-ray diffractometer. The X-ray beam utilized for the analysis was generated by nickel-filtered Cu Kα (λ = 1.542Aº) radiation, operating at 30 kV and 30 mA. All diffractograms were generated within a 2θ range from 5° to 85° at a scanning rate of 1.1/min. Thermogravimetric measurements were conducted using the TGA/DSC1 model from Mettler Toledo, Switzerland, under an inert atmosphere, ranging from room temperature to 800 °C. The heating rate was maintained at 20 °C/min with a nitrogen flow rate of 100 mL/min. All samples underwent vacuum drying prior to SEM analysis and were subsequently coated with amorphous carbon to enhance surface conductivity.

### 2.7. Zeta potential measurements

The zeta potential measurements of PGE_3_-P were conducted using a Zeta Meter 3.0 equipped with a microprocessor unit. The pH-dependent zeta potential variations were investigated. A 0.5 g sample of PGE_3_-P in 100 mL of distilled water, containing the desired electrolytes, was introduced into a thermostatic shaker bath and left to rinse for 24 h at 25 ± 1 °C. Afterward, the samples were allowed to settle for 1 min to facilitate the settling of larger particles. Zeta potential was then measured using an aliquot taken from the supernatant.

### 2.8. Metal removal studies

A stock solution (1000 ppm) of analytical grade CuSO_4_·5H_2_O was prepared in distilled water. The required concentration of metal ions was attained by diluting its stock solutions in water. The mixtures containing the adsorbent and Cu (II) solution were placed in a shaker for a certain time, at 800 rpm. An electronic pH meter was used to examine the pH of the solutions, and the pH was adjusted as necessary with the addition of either 0.1 M HCl or 0.1 M NaOH solution. A Varian SpectrAA-300 atomic absorption spectrophotometer was used to measure the amount of metal ions in liquid samples following the adsorption experiments. Various parameters influencing the adsorption process, such as pH, adsorbent weight, contact time, adsorbate concentration, and temperature, were studied. The adsorption capacity (Q_e_) of the adsorbent was measured using the following equation:


$$\text{Adsorption Capacity}={\text{Q}}_{\text{e}}=\frac{\left({\text{C}}_{\text{o}}-{\text{C}}_{\text{e}}\right)}{\text{m}}\times \text{V}$$

where C_o_ = Initial concentration (mg/L) of metal, C_e_ = Equilibrium concentration of metal, V = Volume (mL) of solution, and m = Mass (mg) of the adsorbent.

## 3. Results and discussion

### 3.1. Free radical graft polymerization

**Figure 2:**
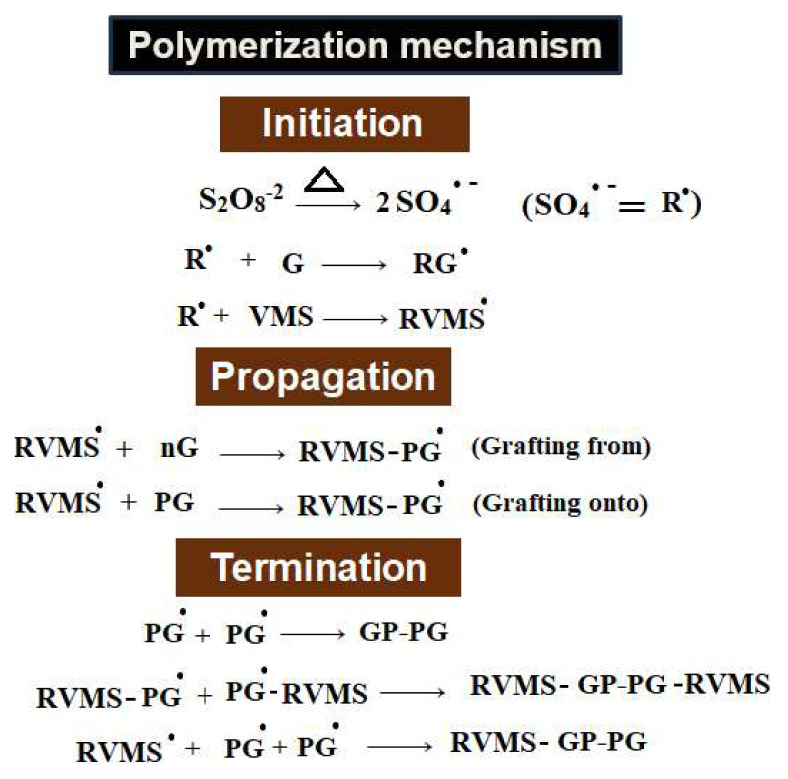
Chemical reactions involved in synthesizing PGE via free radical free radical graft polymerization.

PGMA-grafted sepiolite nanohybrids, synthesized using a free radical graft polymerization method, follow a free radical induced polymerization mechanism. This mechanism consists of initiation, propagation, and termination reactions. The process begins with the dissociation of the initiator (S_2_O_8_^−2^) upon heating, leading to the generation of free radicals (SO_4_^−•^ = R^•^). These free radicals react with GMA monomer molecules (G) and vinyl groups present on VMS to start the graft polymerization reaction as well as homopolymerization of GMA. The growing polymer chains propagate by the interaction of free radicals with other GMA molecules. Since both, the monomer radicals and the VMS, contain vinyl groups, active sites are generated and the grafting process follows “grafting onto” and “grafting from” mechanisms. The propagating polymer chains are terminated by self-reaction of “n” GMA free radicals (nG^•^), leading to homopolymerization, and PGMA grafted radicals (pG^•^-RVMS), which ultimately results in the formation of the grafted polymer product [22]. The chemical reactions involved in the mechanism of free radical graft polymerization of PGMA on VMS is illustrated in Figure 2.

### 3.2. Influence of reaction parameters

The polymerization reaction parameters, including the amount of initiator, monomer concentration, and reaction time, play a significant role in determining the polymer grafting percentageClick or tap here to enter text.. These parameters were optimized to obtain the maximum grafting percentage of PGMA on VMS, and the obtained results are described below.

#### 3.2.1. Effect of initiator amount

**Figure 3:**
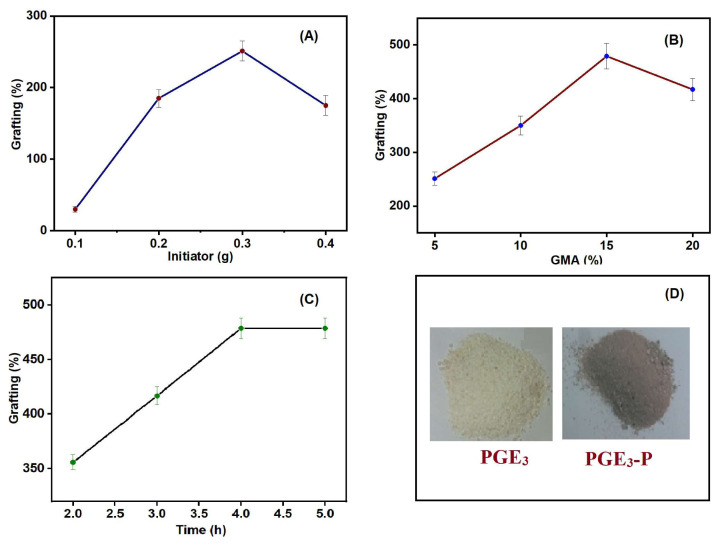
(A) Effect of initiator amount on PGMA grafting percentage: reaction time = 3 h, GMA = 5% (w/v). (B) Effect of monomer concentration on PGMA grafting percentage: reaction time = 3 h, KPS = 0.3% (w/v). (C) Effect of reaction time on polymer grafting percentage: GMA = 15% (w/v), KPS = 0.3% (w/v); VMS = 1.0% (w/v), tween-80 = 0.1% (w/v), T = 70 °C for (A), (B), and (C). (D) Photographs of PGE_3_ and PGE_3_-P nanohybrids.

The effect of the initiator (KPS) on the polymer grafting percentage was thoroughly investigated. The amount of KPS was systematically varied from 0.1% to 0.4% w/v, and the results are presented in Figure 3a. The figure shows that an increase in the grafting percentage was observed with the rise in the amount of KPS. As the KPS amount increases, the grafting percentage also increases. This is because more KPS generates higher number of free radicals for reaction.

Notably, at 0.3% (w/v) initiator amount, an optimal balance was achieved, ensuring an adequate supply of free radicals for both GMA polymerization and grafting reactions. At this point, the maximum grafting percentage of 251% was achieved. However, when the initiator amount was increased beyond 0.3% (w/v), it led to a decrease in the grafting percentage. This decrease was primarily due to a significant increase in the formation of free radicals. The excessive presence of free radicals promoted the homopolymerization of GMA and utilization of larger proportion of the monomer in homopolymerization rather than participating in the grafting reaction, thereby reducing the grafting on the substrate [23].

Based on these results, 0.3% (w/v) KPS amount was fixed for further reactions, as it yielded the best results in terms of grafting efficiency and minimized unwanted homopolymerization.

#### 3.2.2. Effect of monomer concentration

In Figure 3b, the impact of monomer concentration on the grafting percentage of PGMA is depicted. The monomer concentration was changed from 5% to 20% (w/v) and the amount of initiator and surfactant was kept constant. The grafting percentage exhibited a considerable increase with a rise in monomer concentration, reaching its maximum value of 479% at 15% (w/v). However, beyond this point, a rise in the monomer concentration led to a decline in the grafting percentage. This suggests that there is an optimal concentration of monomer for achieving the highest grafting percentage, and exceeding this concentration hinders the grafting reaction, which is 15% (w/v) in our case. At this optimal monomer concentration, the maximum number of GMA molecules can participate in the grafting reaction. However, at a higher monomer concentration (20% (w/v)), the grafting percentage decreased. This can be attributed to the presence of a huge amount of monomer, which led to excessive homopolymerization. The excessive homopolymer formed was physically adsorbed on the VMS surface, creating a barrier that hindered the accessibility of GMA monomers and oligomers. This impediment further hindered the grafting of PGMA onto VMS. As a result, the grafting process was restricted, leading to the observed decrease in grafting percentage at higher monomer concentrations [24]. Based on these results, a GMA concentration of 15% (w/v) was fixed in further experiments.

#### 3.2.3. Effect of reaction time

The impact of reaction duration on the polymer grafting percentage was examined, and the corresponding results are presented in Figure 3c. The reaction time was varied from 2 to 5 h for a specific concentration of reactants, i.e. KPS and GMA. It was observed that as the reaction time increased, a higher grafting percentage was obtained. This rise in grafting percentage can be ascribed to the availability of more free radicals for the graft polymerization reaction, which promotes the polymer chain propagation and growth on VMS. The maximum grafting percentage of 479% was achieved after 4 h. It can be concluded that 4 h was a sufficient duration for the radicals to react and promote efficient polymerization and grafting on the substrate. After 4 h, the grafting percentage showed minimal change. This can be attributed to the consumption of all the reactants during the reaction, and no further alteration in the grafting percentage was observed even with an extended reaction time [25]. Using the optimized conditions and suitable quantities of reactants, a reasonable amount of grafted product was prepared for further studies. The photographs of PGE_3_ and PGE_3_-P, i.e. the synthesized nanohybrids are presented in Figure 3d.

### 3.3. FTIR analysis

**Figure 4:**
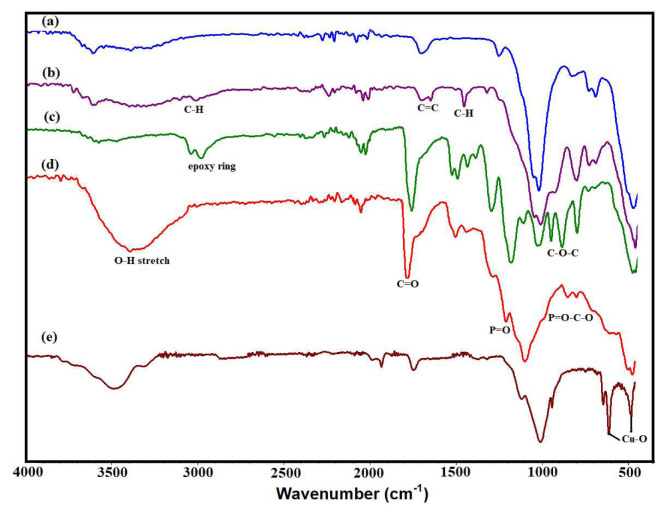
FTIR spectra (a) PS, (b) VMS, (c) PGE_3_, (d) PGE_3_-P, and (e) PGE_3_-P-Cu.

FTIR spectroscopy was used to analyze the chemical changes occurring during the modification, grafting, and phosphonation processes, and the corresponding results are presented in Figure 4. The FTIR spectrum of PS, as shown in Figure 4a, exhibits bands within the 3690–3410 cm^−1^ range, indicative of stretching vibrations attributed to OH groups. These hydroxyl groups suggests the presence of water molecules incorporated within the sepiolite structure. Additionally, the peak detected at 1653 cm^−1^ corresponds to the OH bending of water molecules present in sepiolite. Furthermore, peaks ascribed to the Si-O-Si groups of the tetrahedral sheets of sepiolite were detected at 1004 cm^−1^ and 464 cm^−1^, along with Si-O vibrations at 974 cm^−1^, 783 cm^−1^, 685 cm^−1^ and 644 cm^−1^ [26].

In the IR spectrum of VMS (Figure 4b), new peaks emerged at 2932 cm^−1^ and 2886 cm^−1^, which can be attributed to the asymmetric and symmetric stretch of the –C-H group of the vinyl group present in the sepiolite structureClick or tap here to enter text.. The peaks observed at 1714 cm^−1^ and 1652 cm^−1^ correspond to the C=C stretch of the vinyl group and the OH bending of water molecules present in sepiolite, respectivelyClick or tap here to enter text.. Moreover, the IR spectrum revealed the existence of peaks at 1392 cm^−1^ and 1274 cm^−1^, corresponding to the –C-H bending vibration of vinyl groups present in the sepiolite structure [27].

In the FTIR spectrum of PGE_3_ nanohybrid (Figure 4c), peaks representative of both VMS and GMA are present. It reveals important peaks related to specific functional groups present in the grafted nanohybrid. The peaks at 2996 and 2936 cm^−1^ can be attributed to the stretching vibrations of -CH groups, while their corresponding bending vibrations are observed at 1479 cm^−1^. A noticeable peak at 1721 cm^−1^ was detected, signaling the presence of the carbonyl (–C=O) group in GMA. Additionally, peaks associated with epoxy groups were observed at 1250 cm^−1^, 905 cm^−1^, and 843 cm^−1^. The presence of all peaks of GMA in PGE_3_ spectrum confirms the grafting of PGMA onto VMS. In the FTIR spectrum of PGE_3_-P (Figure 4d), a distinct -OH band emerges in the range of 3500–3000 cm^−1^. Additionally, the peak at 1163 cm^−1^ and 769 cm^−1^ emerged due to the presence of P=O and P-O-C stretchesClick or tap here to enter text.. The presence of these peaks in the spectrum of PGE_3_-P confirms the successful phosphonation reaction. FTIR spectrum of PGE_3_-P-Cu exhibited the peaks at 750 cm^−1^ and 470 cm^−1^ attributed to Cu-O bonds. The presence of these peaks confirms the interaction of PGE_3_-P with Cu (II) ions [28].

### 3.4. XRD analysis

**Figure 5:**
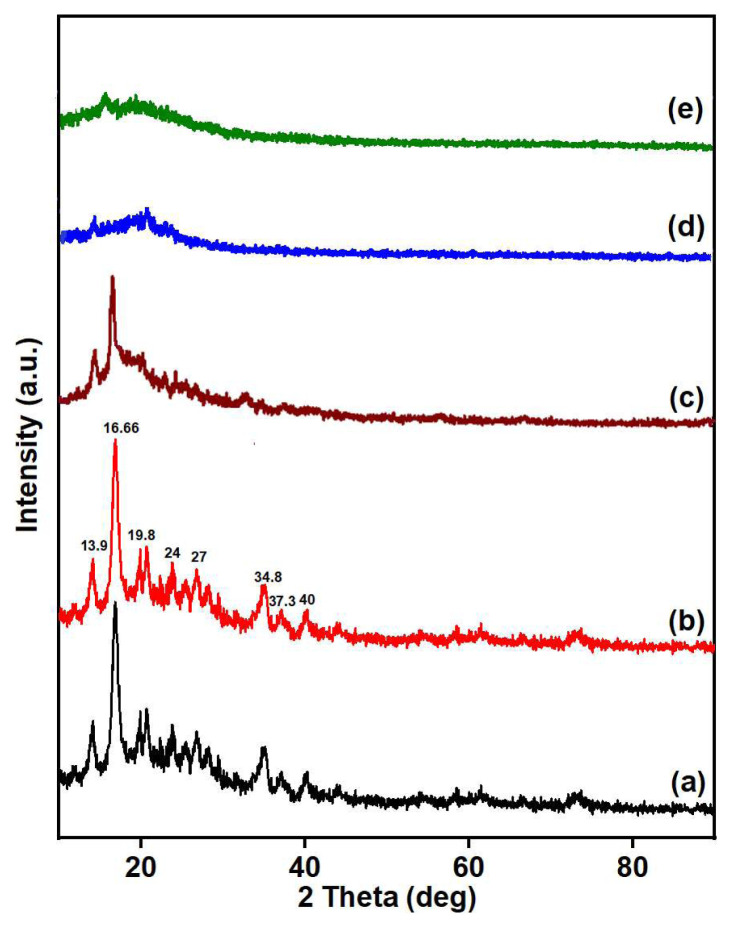
XRD diffractogram of (a) PS, (b) VMS, (c) PGE_3_, (d) PGE_3_-P, and (e) PGE_3_-P-Cu.

The structural analysis of PS, VMS, PGE_3_, and PGE_3_-P was conducted using XRD analysis, and the diffractograms are shown in Figure 5. The diffractogram of PS (Figure 5a) displayed all the characteristic peaks as specified in the standard XRD pattern for pristine sepiolite (JCPDS Card No.: 00-029-1492). These peaks were observed at the following angles: 16.6° (060), 19.8° (131), 20.4° (260), 24°, 27°, 34.8°, 37.3°, and 40° [29]. In the diffractogram of VMS, presented in Figure 5b, a slight decrease in the peak intensities was observed, suggesting that the reaction of VMS with PS slightly affects its structure, but it maintained its structural integrity even after modification.

The spectrum of PGE_3_ (Figure 5c) displayed peaks at 16.6°, 24°, 27°, and 34.8°, which correspond to VMS. However, these peaks exhibited slight positional shifts compared to the standard VMS pattern [30]. The grafting of PGMA on VMS leads to a reduction in crystallinity, indicating a decrease in the overall degree of crystalline ordering. Characteristics peaks of PGMA were observed at 2θ = 17.5° and 2θ = 29.0°. These reflections confirm the presence of grafted PGMA on VMS, indicating the successful grafting of PGMA chains onto VMS. A decrease in the height of peaks and the disappearance of peaks beyond 2θ = 40° indicates the dilution of the crystalline phase of VMS after grafting of amorphous PGMA. However, the average crystallite size increases after PGMA grafting, suggesting a growth or enlargement of individual crystalline domains. The reduced crystallinity of PGE_3_ promotes adsorption, due to increase in amorphous character. The diffractogram of PGE_3_-P, as shown in Figure 5d, depicts a reduced peak intensity compared to PGE_3_. The reduction in intrinsic crystallinity of PGE_3_ was due to incorporation of amorphous organic (PO_3_H) group. The diffractogram of PGE_3_-P-Cu, as shown in Figure 5e, exhibits complete disappearance of the peaks, demonstrating the formation of a chelate between copper ions and PGE_3_-P. Table 2 presents the crystallinity (%) and crystallite sizes of pristine sepiolite and its modified forms.

### 3.5. Morphological analysis

**Figure 6:**
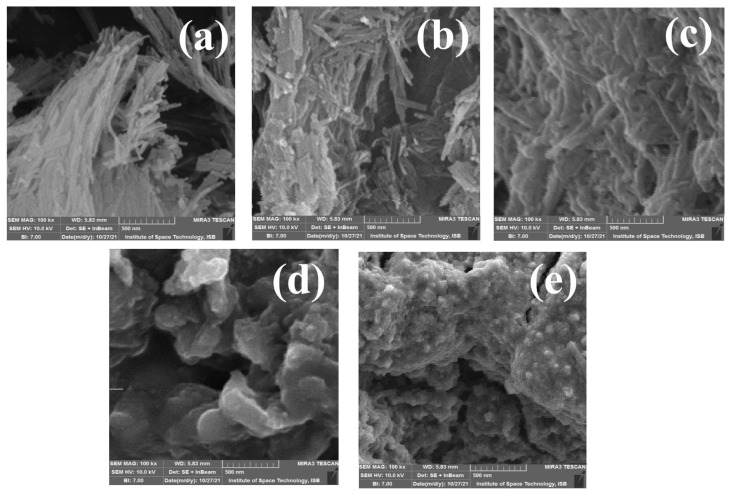
SEM micrographs of (a) PS, (b) VMS, (c) PGE_3_, (d) PGE_3_-P, and (e) PGE_3_-P-Cu.

Figure 6 shows the SEM micrographs of PS, VMS, PGE_3_, and PGE_3_-P nanohybrids. The SEM micrograph of PS (Figure 6a) displays a smooth surface and long bundles of sepiolite fibers. These bundles are formed due to van der Waals forces and hydrogen bonding between adjacent fibers [31]. Upon silanization, as depicted in Figure 6b, the sepiolite fibers become shorter and their bundles are disrupted. This transformation is attributed to the interaction between the silanol groups on the sepiolite fibers’ surface and the vinyl groups of VTMS is responsible for this transformation [32]. The introduction of nonpolar vinyl groups through VTMS treatment also disrupts the electrostatic interactions between the sepiolite fibersClick or tap here to enter text..

In the SEM micrograph of PGE_3_ (Figure 6c), the observed changes include appearance of new surfaces, providing evidence for the growth of PGMA chains on the surface of VMS. As a result of the grafting process, the PGMA chains have fully enveloped the individual fibers of VMS, resulting in a distinct morphology [33]. In contrast to the well-defined surface of PS, PGE_3_ possesses a rough surface with tightly attached embedded fibers. The results confirm the successful grafting of PGMA on VMS surface. The micrographs of VMS and PGE_3_ are in agreement with the XRD analysis as the structural integrity of sepiolite was maintained in VMS and PGE_3_ because functionalization occurred largely on the surface or by partial replacement of zeolitic waterClick or tap here to enter text..

Phosphonation of PGE_3_ resulted in a further increase in surface roughness and compactness, as depicted in Figure 6d. This effect can be attributed to the hydrophilic nature of the introduced phosphonic acid groups, as reported by previous studiesClick or tap here to enter text.. FESEM micrographs of PGE_3_-P also revealed the presence of globular and compact structures. Notably, the fibers of PS and VMS were entirely covered with PO_3_H groups, indicating successful phosphonation of the surface [34]. Figure 6e displays the change in morphology of PGE_3_-P after Cu (II) adsorption. The image indicates that after Cu (II) adsorption, the size of globules further increases.

### 3.6. CHO analysis

CHO analysis was performed to examine the alterations in the composition of PS during its silanization and grafting process. Table 3 shows the results of elemental analysis of the sepiolite. The detection of carbon in the CHO analysis of VTMS provides evidence supporting the silanization and incorporation of vinyl groups onto the PS surface during this reaction. Moreover, a decrease in the atomic percentage of oxygen occurred due to the silanization reaction between VTMS and sepiolite. In this reaction, the silanol groups of sepiolite reacted via a condensation reaction with VTMS, resulting in the removal of water molecules occurs. Consequently, the oxygen content decreased in VMS.

The atomic percentage of carbon was further increased in PGE_3,_ which can be attributed to the PGMA grafting onto the PS. PGMA grafting introduces additional carbon atoms to the VMS structureClick or tap here to enter text.. Additionally, the oxygen atomic percentage increases in PGE_3_, which is due the oxygen of epoxy and carbonyl groups of grafted PGMA present in PGE_3_Click or tap here to enter text.. CHOP analysis of PGE_3_-P shows an increase in atomic percentages of oxygen and hydrogen compared to PGE_3_, and the presence of P percentage confirms the successful incorporation of phosphonic acid group in the structure of PGE_3_ and its conversion into PGE_3_-P.

These results from the elemental analysis provide valuable insights into the compositional variations that occur during synthesis of PS nanohybrids.

### 3.7. BET analysis

Chemical changes significantly affect the surface area, pore volume, and pore size of a material. The influence of chemical treatments on PS and its modified forms was assessed using BET analysis. The findings of this analysis are outlined in Table 4. It is evident from the table that a noticeable decrease in the surface area, pore volume, and pore size occurred in PS during the silanization reaction. This condensation reaction introduced new functionalities to sepiolite, leading to a significant decrease of approximately 75.5% in its surface area. The grafted sample, namely PGE_3_, exhibited an even more reduced surface area, pore volume, and pore size. Grafting PGMA chains filled the pores on the VMS surface. This grafting reaction resulted in reduction of up to 64% in pore volume and approximately 40% in pore size. The BET analysis results indicated that the silanization and subsequent grafting reaction significantly influenced the surface area, pore size, and pore volume of PS. It is important to note that these are crucial factors in a material’s properties and its applications.

The surface area of the PGE_3_ decreased even further after phosphonation because of the incorporation of phosphonic acid groups on PGE_3_. Additionally, a reduction in pore volume and pore size was observed after phosphonation, attributed to the filling of empty pores with bulky polymeric chains containing PO_3_H groups at the surface. These filled pores are also visible in the SEM micrographs provided in Figure 6.

### 3.8. Thermogravimetric analysis

**Figure 7:**
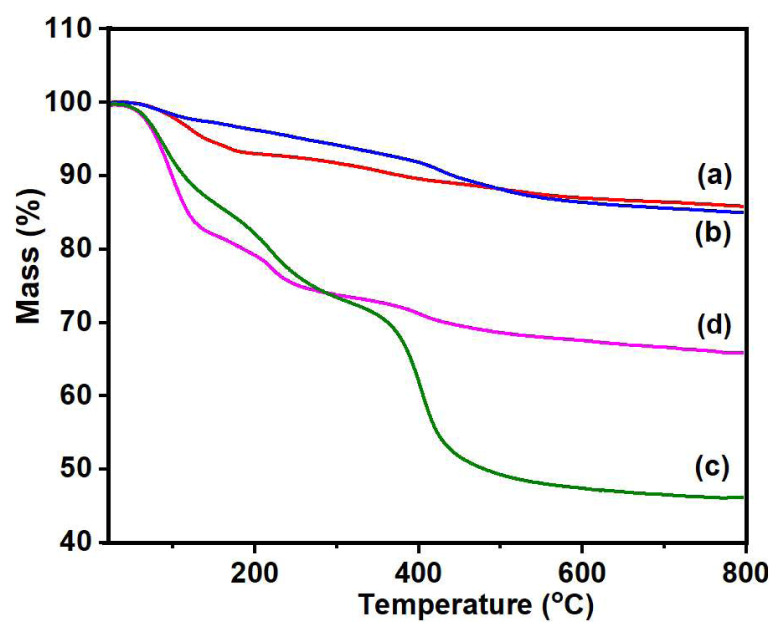
TGA thermogram of (a) PS, (b) VMS, (c) PGE_3_, (d) PGE_3_-P.

Thermal stability of the synthesized nanohybrids was assessed using TGA, and the corresponding thermograms are presented in Figure 7. The thermogram of PS (Figure 7a) showed a three-step mass loss. In the first step (58–172 °C), an 8% mass loss was detected because of the removal of adsorbed water on sepiolite [35]. Subsequently, from 172 to 395 °C, a 4% mass loss was observed as a result of the loss of zeolitic and coordinated water [36]. In the third step, up to 580 °C, a 2.5% mass loss was observed, attributed to the dehydroxylation of sepiolite anhydride [37]. The thermogram of VMS (Figure 7b) demonstrated an approximately 2.5% mass loss from 50 to125 °C due to loss of the adsorbed water, followed by another 2.5% mass loss occurring from 125 to 240 °C due to the removal of zeolitic and coordinated water Click or tap here to enter text.. Decomposition of vinyl groups occurred from 240 to 550 °C with a 10% mass loss [38].

The thermogram of PGE_3_ (Figure 7c) showed a 14.5% mass loss up to 58.5–140 °C ascribed to the removal of adsorbed waterClick or tap here to enter text.. In the next step, a 12% mass loss was observed up to 140–240 °C, which could be attributed to the removal of physiosorbed water Click or tap here to enter text., or water adsorbed to the material’s surface through van der Waals forces or hydrogen bonding Click or tap here to enter text.. The degradation of grafted PGMA chains starts from 240 °C and a 33.5% mass loss can be seen at 550 °C. This decomposition could result in the depolymerization of PGMAClick or tap here to enter text. or the formation of glycidyl, allyl alcohol, isobutene, CO, CO_2_, acrolein, and propene through ester degradation [39].

The thermogram of PGE_3_-P (Figure 7d) reveals an initial step of mass loss, amounting to 19.6%, occurring within the temperature range of 52 °C to 134 °C. This mass loss is attributed to the evaporation of water molecules linked to the hydrophilic PO_3_H groups A further mass reduction of 8.6%, occurring between 134 and 240 °C can be ascribed to the removal of coordinated water from sepiolite. A mass loss of 8.1%, occurring from 240–550 °C can be attributed to the degradation of the grafted PGMA chains [40]. The thermal stability of PGE_3_-P surpasses that of its unsulfonated form, and this increased stability can be attributed to the phosphonation process [41]. The remaining residues consist mainly of the char resulting from the carbonaceous polymeric material and clay silicates [42]. The residual mass of both pristine sepiolite and its modified forms at 800 °C is provided in Table 5.

## 4. Postpolymerization modification of PGMA grafted sepiolite nanohybrids

GMA is a vinyl monomer characterized by the presence of an epoxide group. The distinctive reactivity of the epoxy group allows for a wide range of reaction pathways. For instance, when PGMA reacts with phosphoric acid or other acids, a ring-opening reaction takes place. The unique characteristic of epoxide polymers enables a broad spectrum of functionalization possibilities under mild reaction conditions. As a result, GMA and its derived polymers provide significant flexibility in tailoring the properties of polymer adsorbents to meet specific application requirements [43].

In the present work, the maximum grafted product, PGE_3_, was utilized as a precursor to create phosphonic acid-functionalized grafted sepiolite. This was achieved by reacting PGE_3_ with H_3_PO_4_ through the ring opening reaction of the epoxy group present in PGMA [44].

### 4.1. Cu adsorption on phosphonic acid-functionalized grafted sepiolite nanohybrids

The phosphonic acid-functionalized grafted sepiolite nanohybrid (PGE_3_-P) was employed for the adsorption of Cu (II) ions from aqueous solutions. Cu (II) was selected as a model pollutant due to the presence of -PO(OH)_2_ groups in PGE_3_-P, which exhibit a higher affinity for Cu (II) ions compared to other metal ions. The chemical nature of the phosphonic acid groups enables it to form stronger coordination bonds with Cu ions [45]. The results of adsorption studies obtained by varying the pH, adsorbent weight, contact time, adsorbate concentration and temperature are presented below.

#### 4.1.1. Effect of pH

**Figure 8:**
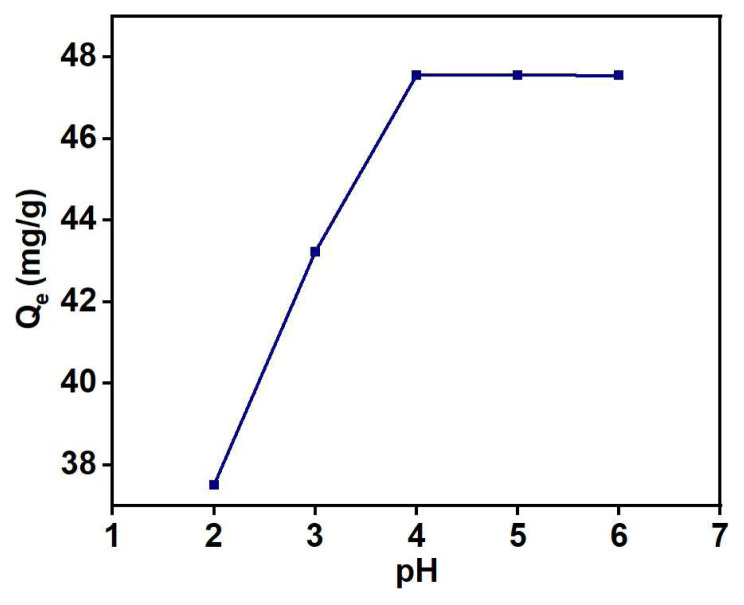
Effect of pH on Cu (II) ions uptake: adsorbent weight = 4 g/L, time = 1 h, C_o_ = 20 mg/L and T = 398 K.

To study the effect of pH on Cu (II) adsorption by PGE_3_-P nanohybrid, the pH of the aqueous solution was changed from 2 to 6. It should be noted that pH influences the adsorption process by altering the surface charge and speciation of metal ions. Figure 8 illustrates a linear increase in Cu (II) adsorption capacity with with rise in pH up to 4. At low pH levels, the reduced adsorption of Cu (II) ions is attributed to the competition between hydrogen ions and metal ions for binding with the phosphonic acid groups. Additionally, the oxygen groups present in PGE_3_-P become more protonated at low pH values, rendering them less accessible to effectively capture Cu (II) ions [46].

At 4 pH, the maximum adsorption capacity was observed. This enhanced adsorption capability is ascribed to the presence of free lone pairs of electrons on deprotonated oxygen atoms within the PGE_3_-P structure. These exposed lone pairs serve as appropriate ligands for coordinating with the copper ions, facilitating stronger and more effective adsorption interactions [47]. At higher pH values, adsorption capacity decreases because Cu (II) ions tend to precipitate as hydroxides. Based on the obtained results, the pH of the aqueous solution was kept 4 in further experiments.

**Figure 9:**
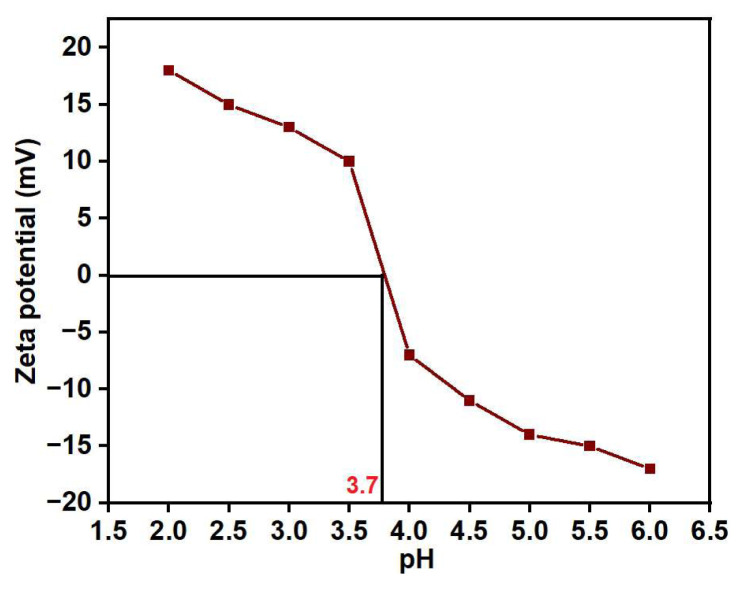
Zeta potential of PGE_3_-P at pH ranging from 2 to 6.

The pH dependence of the zeta potential in PGE_3_ is also notable. As the pH increases from 2 to 6, the values of zeta potential decreases progressively from 18.1 to −17.9 mV. The pH of the zero-point charge (pH_ZPC_) was observed at 3.7 Zeta potential of PGE_3_-P at pH ranging from 2 to 6 is depicted in Figure 9.

#### 4.1.2. Effect of adsorbent weight

**Figure 10:**
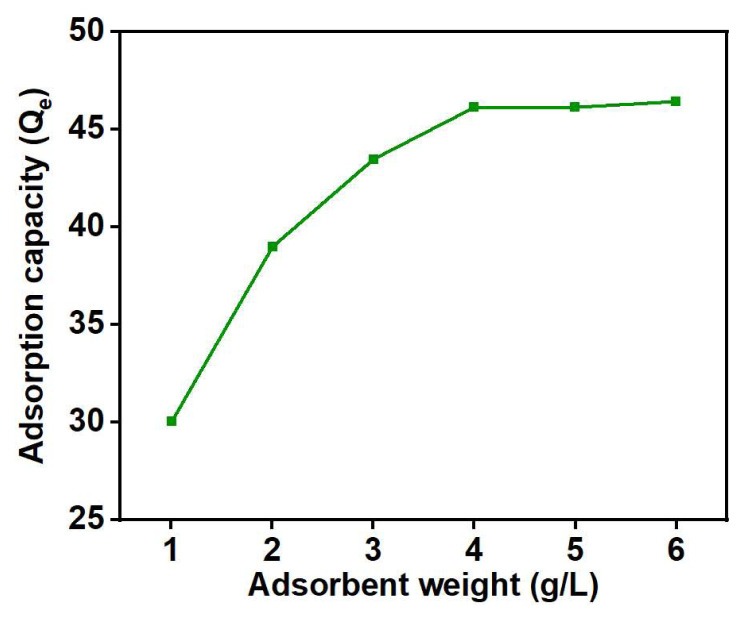
Effect of adsorbent weight on Cu (II) uptake: pH = 4, time = 1 h, C_o_ = 20 mg/L, and T = 398 K.

The weight of the adsorbent is also a pivotal parameter in metal adsorption studies, and it can significantly affect the efficiency of an adsorption process. If the adsorbent weight is too low, the active sites may not be available or limited, resulting in incomplete capture of metal ions in the solution. Conversely, excessive or overly high weight of the adsorbent can complicate the separation or recovery process of the nanohybrid from the solution, thus increasing the cost [48]. Hence, optimizing the weight of the adsorbent is crucial to achieve optimal efficiency and cost-effectiveness. The adsorbent weight was varied from 1 to 6 g/L in a metal adsorption process to investigate its influence on the adsorption of Cu (II) from aqueous solutions and the results are shown in Figure 10. A rise in Cu (II) adsorption capacity can be observed, ranging from 30 mg/g to 46 mg/g, as the adsorbent weight increases from 1 g/L to 4 g/L. Increasing the weight of adsorbent can elevate the number of accessible adsorption sites. Moreover, as the adsorbent weight increases, the likelihood of copper ions or molecules interacting and adhering to the adsorbent material enhances, resulting in increased adsorption capacity for Cu (II) [49].

After reaching 4 g/L, further increases in adsorbent weight do not notably affect the adsorption process. This can be attributed to the limited number of adsorbate molecules at this point. Although an excess of adsorbent is available in the solution, there are no more adsorbate molecules available for adsorption at this stage [50]. Based on these results, an adsorbent weight of 4 g/L was fixed for further studies.

#### 4.1.3. Effect of contact time

**Figure 11:**
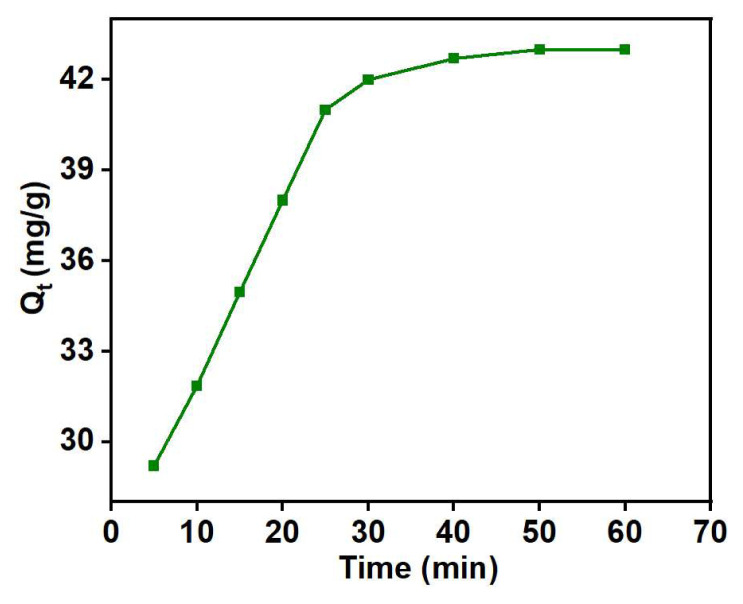
Effect of contact time on Cu (II) uptake: pH = 4, adsorbent weight = 5 g/L, C_o_ = 20 mg/L, and T = 398 K.

In an adsorption process, optimizing contact time is crucial to achieve the maximum adsorption capacity. Contact time serves as a vital parameter for exploring the kinetics of the adsorption process. Figure 11 illustrates the impact of contact time intervals on the adsorption capacity of Cu (II). The adsorption capacity exhibits a gradual increase with extended contact time. This behavior is attributed to the abundance of available adsorption sites on the surface of the adsorbent, enhancing the reaction rate [51]. After 50 min, no significant increase in adsorption capacity is observed. This is because, at this stage, the adsorption sites on the adsorbent surface become saturated [52], reaching their maximum capacity where additional adsorbate molecules cannot be accommodated [53]. Based on these results, a contact time of 50 min was fixed for further studies.

**Figure 12:**
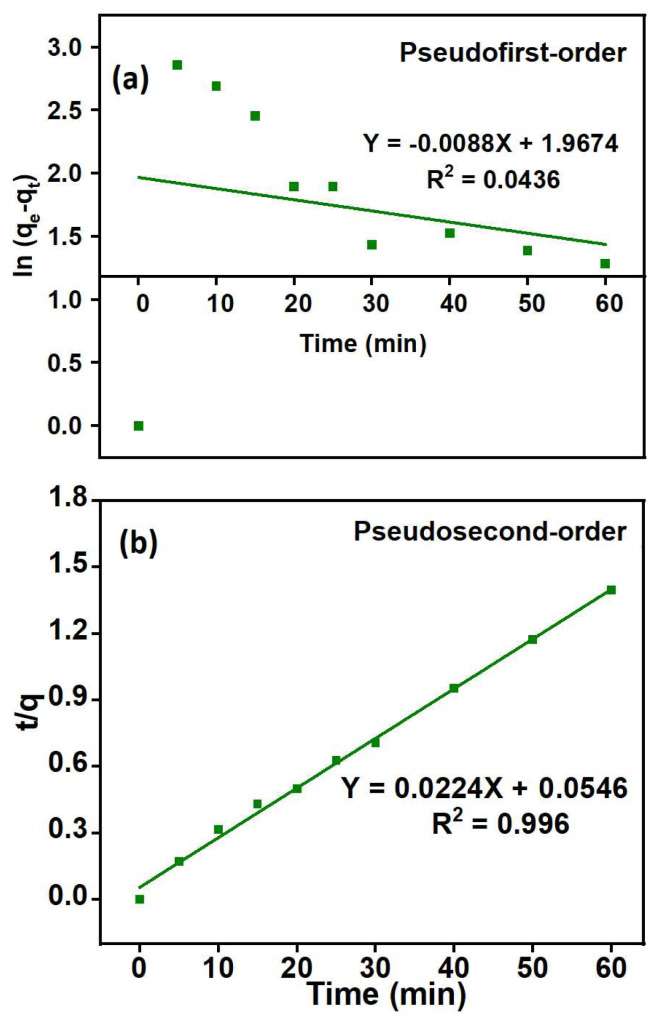
(a) Pseudofirst-order and (b) pseudosecond-order plots of Cu (II) adsorption kinetics on the PGE_3_-P: pH = 4, adsorbent weight = 5 g/L, C_o_ = 20 mg/L, and T = 398 K.

To investigate the adsorption kinetics, pseudofirst-order and pseudosecond-order models were applied and the results are presented in Figure 12. The pseudosecond-order model exhibited a better fit to the experimental data, as evident by higher R^2^ values of 0.997 for PGE_3_-P. The best fitting of pseudosecond-order model suggests that the adsorption process involves the formation of chemical interactions between the adsorbent and adsorbate interface [54]. Table 6 summarizes the comparison of parameters obtained from the kinetic models.

#### 4.1.4. Effect of adsorbate concentration

**Figure 13:**
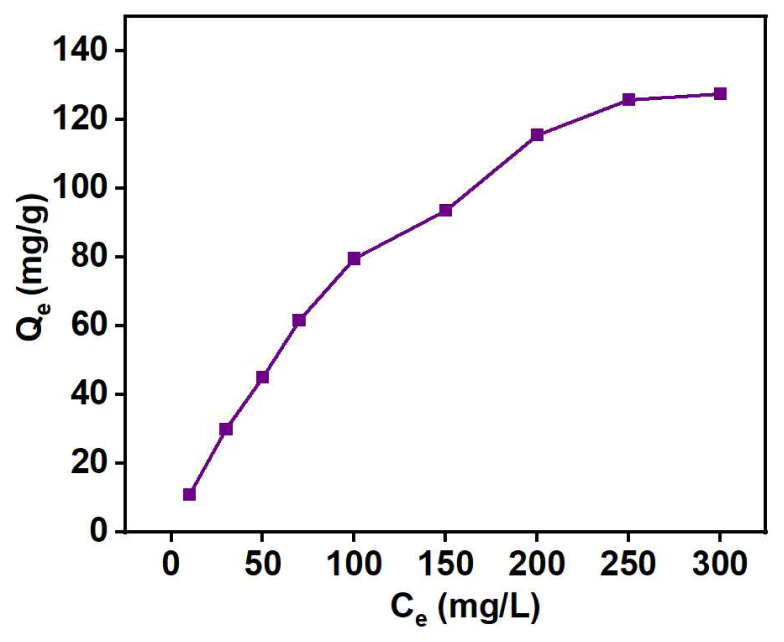
Effect of adsorbate concentration on the Cu (II) uptake: pH = 4, adsorbent weight = 5 g/L, contact time = 50 min, and T = 398 K.

The concentration of adsorbate influences the adsorption capacity, making it essential to optimize its concentration. By finding the right balance in adsorbate concentration, we can achieve the maximum adsorption capacity, leading to potential cost optimization. Figure 13 depicts the impact of adsorbate concentration on the adsorption capacity.

As the initial concentration of Cu (II) ions rises, there is a corresponding increase in the adsorption capacity. Initially, this increase follows a linear trend (up to 100 mg/L), which then becomes more gradual, likely due to a limited amount of available adsorption sites on the adsorbent’s surface [55]. The maximum capacity was observed at 250 mg/L, which was consequently fixed in the next experiment.

**Figure 14:**
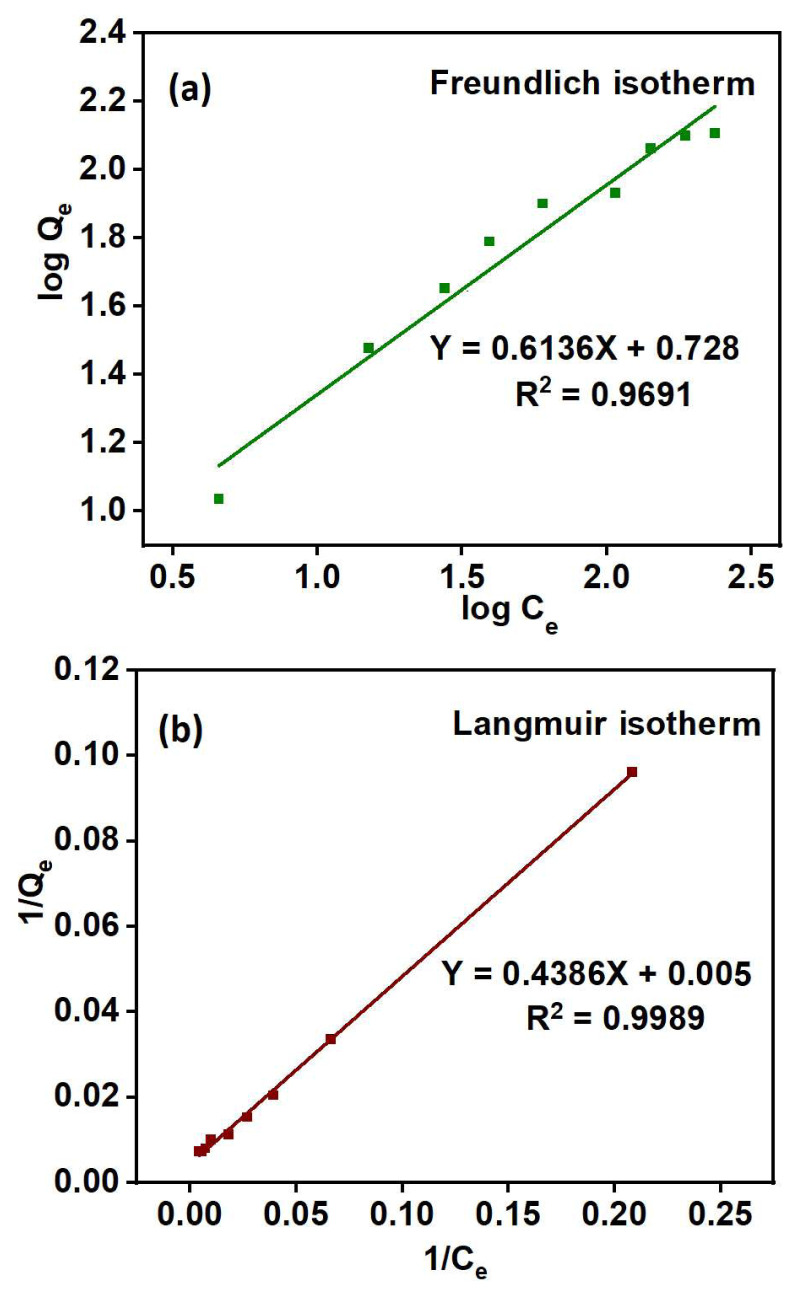
(a) Freundlich and (b) Langmuir model fit for Cu (II) uptake by PGE_3_-P adsorbent: pH = 4, adsorbent weight = 5 g/L, contact time = 50 min, and T = 398 K.

To study the adsorption mechanism, the isothermal data was fitted with the Langmuir [56] and Freundlich [57] isothermal models, and their equations are given below:


$$\begin{array}{l} \frac{{\text{C}}_{\text{e}}}{{\text{Q}}_{\text{e}}}=\frac{1}{{\text{K}}_{\text{L}}{\text{Q}}_{\text{m}}}+\frac{{\text{C}}_{\text{e}}}{{\text{Q}}_{\text{m}}}\;\;\;(\text{Langmuir equation})\\ {\text{logQ}}_{\text{e}}=\text{log}\;{\text{K}}_{\text{F}}+\frac{1}{\text{n}}\text{log}\;{\text{C}}_{\text{e}}\;\;\;(\text{Freundlich equation}) \end{array}$$

where Q_e_ is the adsorption capacity (mg/g) at equilibrium and C_e_ is the equilibrium concentration (mg/L) of metal in solution. K_L_ is the Langmuir constant (L/mg) associated with the adsorption free energy. K_F_ and 1/n are Freundlich and heterogeneity constants (L/g), where K_F_ indicates the extent of adsorption and 1/n indicates the adsorption intensity [58]. As illustrated in Figure 14 and summarized in Table 7, the Langmuir model exhibited a higher R^2^ (correlation coefficient), indicating that the adsorbent PGE_3_-P follows a monolayer [59] adsorption phenomenon for Cu (II) uptake on a homogenous surface [60].

#### 4.1.5. Effect of temperature

**Figure 15:**
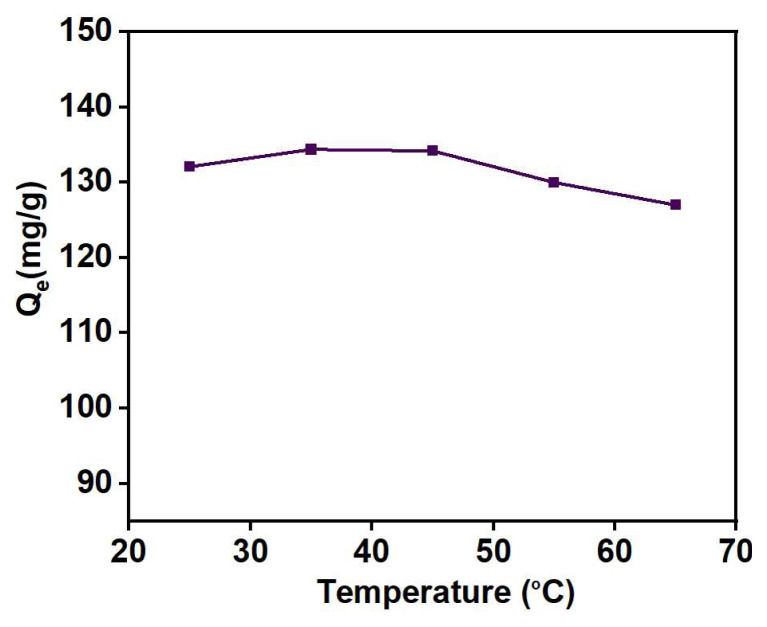
Effect of temperature on the Cu (II) uptake: contact time = 50 min, pH = 5, adsorbent weight = 5 g/L, C_o_ = 250 mg/L.

In an adsorption process, temperature has a significant impact on the adsorption capacity kinetics, as well as the thermodynamics of the process. Figure 15 depicts the results of adsorption studies carried out for Cu (II) uptake at various temperatures. As the temperature increases from 25 to 45 °C, there is a corresponding increase in the adsorption capacity of Cu (II) up to 2%. This rise in temperature imparts greater kinetic energy to molecules, thereby enhancing diffusion rates and facilitating the transport of metal ions from the solution to the adsorbent’s adsorption sites. However, after 45 °C, the adsorption capacity begins to decline, which can be attributed to an increase in the rate of desorption. At higher temperatures, desorption becomes the dominant phenomenon rather than adsorption [61].

Thermodynamic studies provide information about the free energy, enthalpy, and entropy of the system during an adsorption process. Below are the equations utilized to calculate the Gibbs free energy (ΔG◦), enthalpy, and entropy changes (ΔH◦ and ΔS◦) [62].


$$\begin{array}{l} \Delta \text{G}=-\text{RT ln k}\\ \text{ln K}=-\frac{\Delta {\text{H}}^{{}^{\circ}}}{\text{R}}+\frac{\Delta {\text{S}}^{{}^{\circ}}}{\text{R}} \end{array}$$

where R is the universal gas constant (8.314 Jmol^−1^ K^−1^), T is the temperature in kelvin, and K is the equilibrium rate constant. K can be calculated using the following equation:


$$\text{K}=\frac{{\text{C}}_{\text{ads}}}{{\text{C}}_{\text{eq}}}$$

Here C_ads_ is the concentration of adsorbate (Cu (II) ions) in mg/L, adsorbed at surface per liter of the metal solution, C_eq_ is the concentration of metal ions (mg/L) at equilibrium.

**Figure 16:**
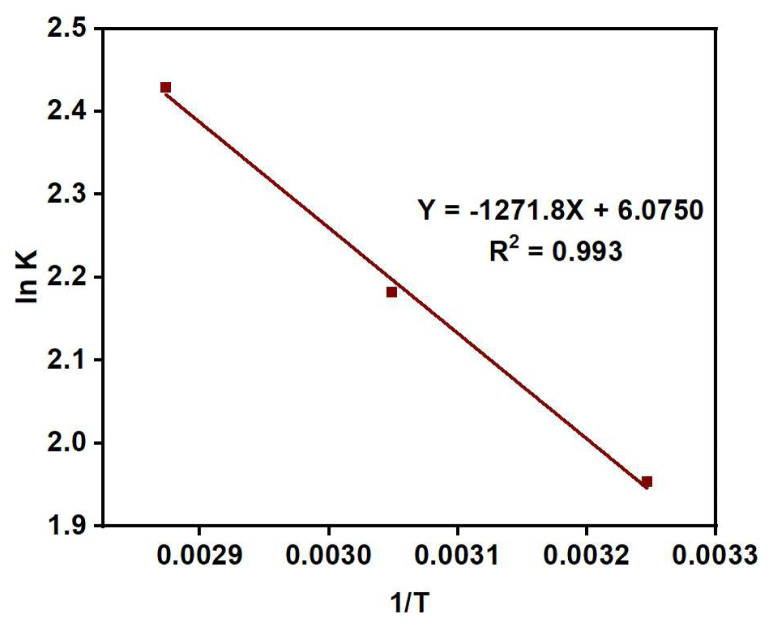
Plot of ln K vs 1/T for calculation of the adsorption thermodynamic parameters.

As presented in Table 8, ΔG° values are consistently negative across all studied temperatures, indicating the spontaneous nature and feasibility of Cu (II) ion adsorption on the adsorbents. ΔG^o^ values falling within the range of −20 to −40 kJ gmol^−1^ suggest electrostatic interactions between sorption sites and metal ions (indicative of physical adsorption), whereas ΔG^o^ values more negative than −40 kJ gmol^−1^ imply charge sharing or transfer from the adsorbent surface to the metal ion, forming a coordinate bond (reflective of chemical adsorption). In this study, the obtained ΔG^o^ values are less than −20 kJ gmol^−1^, indicating that the Cu (II) ion adsorption follows a physical adsorption mechanism at the given temperatures Click or tap here to enter text.. Moreover, the value of ΔH^o^ obtained from the slope of plot of ln K values against 1/T (Figure 16) is positive, which verifies adsorption in this study as an endothermic process. Typically, ΔH values ranging from 2 kJ/mol to 30 kJ/mol indicate physical adsorption, while for chemical adsorption, ΔH^o^ values range from 40 kJ mol^−1^ to 200 kJ mol^−1^ [63]. Hence, the estimated value of ΔH^o^ (10.47 kJ mol^−1^ for PGE_3_-P) demonstrates the dominance of physisorption mechanism in the adsorption process. The positive value of ΔS^o^ (50.51 kJ/mol) indicates that some structural variations occurred at the solid-liquid boundary, leading to increased system randomness [64].

#### 4.1.6. Mechanism of Cu (II) adsorption

**Figure 17:**
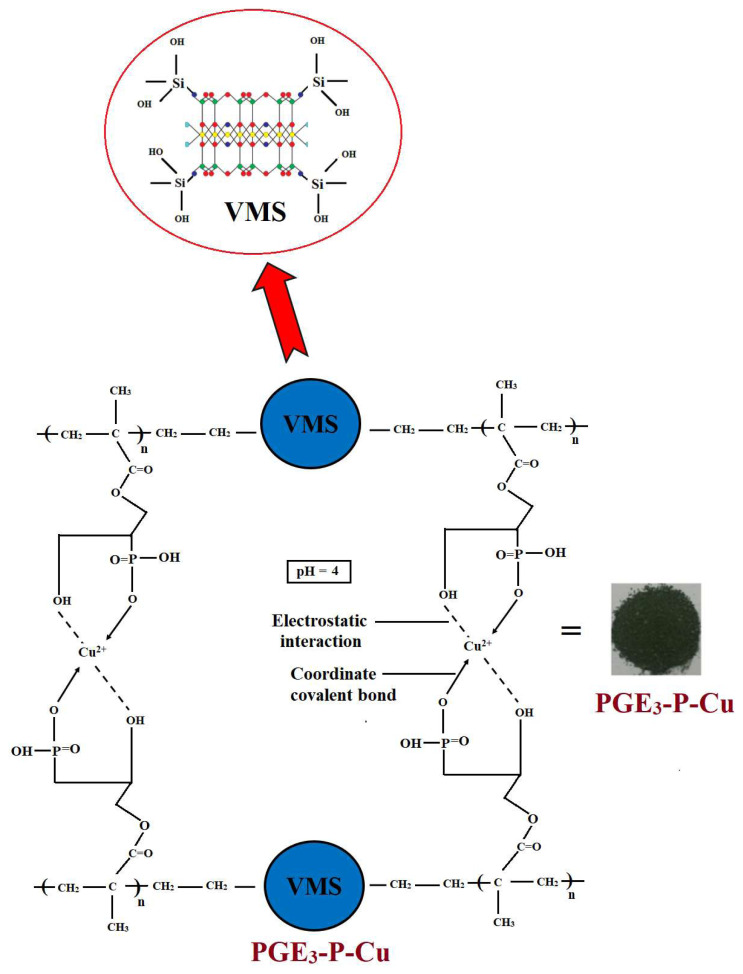
Proposed mechanism of Cu (II) adsorption by PGE_3_-P.

In Cu (II) adsorption, a combination of ion exchange, chemical bonding, electrostatic interactions, complexation, and physical adsorption is involved. Understanding these mechanisms is crucial for designing effective adsorbents for copper removal from polluted water. Often, adsorption kinetic studies and infrared spectroscopic analyses are used to investigate the reaction mechanism in detail. In the present study, the best fit of pseudosecond-order model suggests that adsorption is chemisorption, whereas thermodynamic parameters support physisorption and electrostatic interactions between PGE_3_-P adsorbent and Cu (II) ions. Additionally, the presence of Cu-O peaks in the infrared also supports chemisorption and the formation of covalent bonds between Cu and adsorbent molecules. Thus, from these results, we can conclude that adsorption in the present work is a physio-chemical process, not pure physisorption or chemisorption. Figure 17 illustrates the proposed mechanism of Cu (II) adsorption by PGE_3_-P, based on the conclusions above.

#### 4.1.7. Selectivity and reusability studies

The selectivity of the nanohybrid is demonstrated through adsorption tests conducted in the presence of other metal ions that may potentially interrupt the adsorption process. Here, selectivity tests for PGE_3_-P were carried out in the presence of copper, lead, nickel, and cobalt (100 ppm). The findings are presented in Table 9. The nanohybrid demonstrated distinct selectivity towards Cu (II) ions and effectively resisted interference from other coexisting metal ions.

**Figure 18:**
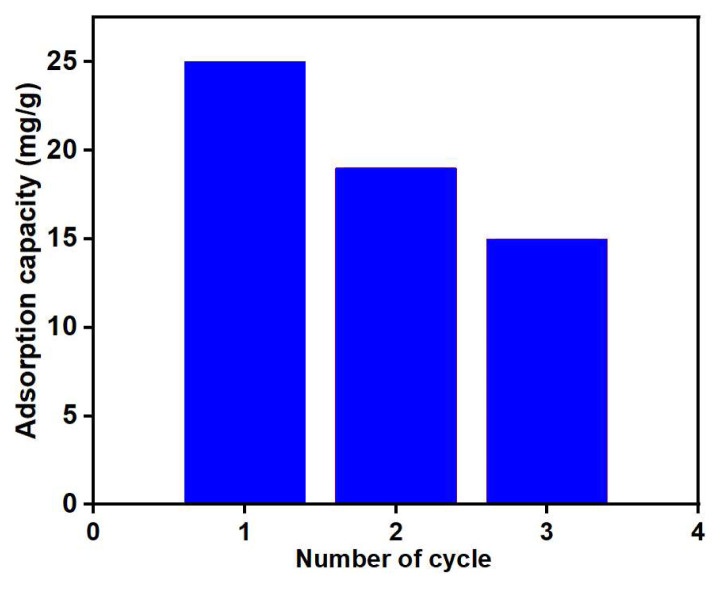
Regeneration studies on PGE_3_-P: adsorbent weight = 5 g/L, V = 20 mL, T = 398 K, desorption medium = NaCl.

The reusability assessments were conducted using 1 M NaCl as the desorption medium, and the outcomes are depicted in Figure 18. The nanohybrid exhibited 68% Cu (II) adsorption capacity over three consecutive adsorption-desorption cycles, relative to the first cycle.

In Table 10, a comparison of the maximum adsorption capacity of different adsorbents for Cu (II) ions reported in the literature has been included. PGE_3_-P exhibited superior adsorption capacity for lead adsorption as compared to other adsorbents.

## 5. Conclusion

In the present study, novel and efficient phosphonic acid-functionalized grafted sepiolite nanohybrids were synthesized via free radical graft polymerization for the selective removal of copper ions from water. Free radical graft polymerization is a cost-effective, easy-to-use, and green process. Moreover, higher grafting percentages of grafted polymers can be obtained with very small amounts of initiators by using this process. Optimization of grafting parameters yielded a maximum grafting percentage of 479% at 15% GMA using 0.3 g initiator in a 4-h reaction. The selected nanohybrid, PGE_3_, underwent further modification by transforming epoxy groups into phosphonic acid groups to enhance Cu (II) ion adsorption capacity. Confirmation of grafting and phosphonation was achieved through FTIR, XRD, and TGA analyses. The presence of new vibrations and changes in crystallinity were observed, and FESEM provided insights into morphological alterations. The BET technique assessed surface area changes in the synthesized nanohybrids.

In adsorption studies, a batch adsorption system was employed to investigate the effects of pH, adsorbent weight, adsorbate concentration, contact time, and temperature. The maximum adsorption capacity of PGE_3_-P adsorbent was found to be 134.5 mg/g for Cu (II) ions. In the kinetics studies, the pseudosecond-order model provided the best fit, while in the equilibrium isothermal studies, the Langmuir isotherm model fitted best. The thermodynamic parameters indicated that the adsorption process is both endothermic and spontaneous in nature.

## Figures and Tables

**Table 1:** Chemical compositions and codes of PGMA-grafted nanohybrids.

Sample code	GMA (w/v %)	KPS (g)	Reaction time (h)
**PGE** **_1_**	5.0	0.3	4.0
**PGE** **_2_**	10.0	0.3	4.0
**PGE** **_3_**	**15.0**	**0.3**	**4.0**
**PGE** **_4_**	20.0	0.3	4.0
**PGE** **_5_**	15.0	0.1	4.0
**PGE** **_6_**	15.0	0.2	4.0
**PGE** **_7_**	15.0	0.4	4.0
**PGE** **_8_**	15.0	0.3	3.0
**PGE** **_9_**	15.0	0.3	5.0
**PGE** **_10_**	15.0	0.3	6.0

* Amount of VMS = 1.0% (w/v); amount of tween-80 = 0.1 % (w/v).

**Table 2:** Crystallinity (%) and average crystallite size (nm) of pristine sepiolite and its modified forms.

Sample codes	PS	VMS	PGE_3_	PGE_3_-P	PGE_3_-P-Cu
**Crystallinity (%)**	83.2 ± 1.07	78 ± 3.07	62 ± 2.07	47 ± 1.01	46.1 ± 1.21
**Average crystallite size (nm)**	106.2 ± 9.07	108.2 ± 9.07	113.2 ± 8.07	153.5 ± 9.05	155.1 ± 7.04

**Table 3:** Comparison of elemental composition of pristine sepiolite and its modified forms.

Sample codes	Atomic percentage			

	C	O	H	P

PS	-	3.5	58.2	-
VMS	55.3	40.8	52.1	-
PGE_3_	67.3	52.7	78.5	-
PGE_3_-P	67.2	65.7	81.2	4.15

**Table 4:** BET surface area analysis of pristine sepiolite and its modified forms.

Sample codes	PS	VMS	PGE_3_	PGE_3_-P
**BET surface area (m** **^2^** **/g)**	212.6	160.8	135.2	93 ± 0.58
**Pore volume (cm** **^3^** **/g)**	59.3	57.1	24.2	19.5
**Pore size (nm)**	62.8	62.4	57.2	68.2

**Table 5:** The residual mass of pristine sepiolite and its modified forms at 800 °C.

Sample codes	Residue (%) at 800 °C
PS	83.8
VMS	82.97
PGE_3_	38.6
PGE_3_-P	61.5

**Table 6:** Comparison of parameters obtained by using pseudofirst-order and pseudosecond-order models for Cu (II) uptake.

Pseudofirst-order				Pseudosecond-order			
R^2^	K_1_ (min^−1^)	Q_e_ (cal) (mg/g)	Q_e_ (exp) (mg/g)	R^2^	K_2_ (g mg^−1^ min^−1^)	Q_e_ (cal) (mg/g)	Q_e_ (exp) (mg/g)
0.0436	−0.00014	7.15	43.1	0.997	0.00919	44.64	43.1

**Table 7:** Comparison of parameters obtained by using the Langmuir and Freundlich models on adsorbents at 398 K.

Freundlich isotherm constants			Langmuir isotherm constants			
1/n	K_F_ (g/mg min^−1^)	R_F_^2^	R_L_^2^	K_L_ (L/mg)	Q_m_ (cal) (mg/g)	Q_m_ (exp) (mg/g)
0.613	2.0718	0.02102	0.02710	0.01015	134.228	132.50

**Table 8:** Thermodynamic parameters for adsorption of Cu (II) on PGE_3_-P.

Temperature (K)	ΔG° at 308	ΔG° at 328	ΔG° at 348	ΔH° (kJ/mol)	ΔS (kJ/mol)
PGE_3_-P	−5.0017	−5.9496	−7.0001	10.4737	50.51

**Table 9:** Effect of other metal ions on adsorption of Cu (II) on PGE3-P.

Element	Cu	Pb	Ni	Co
Adsorption capacity (mg/g)	84.3	24.2	15.2	10.4

**Table 10:** Comparison of adsorption capacities of different materials for Cu (II) ions removal.

Adsorbents	Adsorption capacity (mg/g)	References
Chitosan composites with amidoxime-grafted graphene oxide	115.3	[56]
Chitosan crosslinked with glutaraldehyde/methylene bisacrylamide	95.7	[65]
Poly(acrylic acid)/alginate	63.59	[66]
PGE_3_-P	134.5	Present work
